# “I Like it Clean”: Brazilian Waxing and Postfeminist Subjectivity Among South Asian Beauticians in London

**DOI:** 10.3389/fsoc.2021.646344

**Published:** 2021-06-07

**Authors:** Nandita Dutta

**Affiliations:** Centre for Multidisciplinary and Intercultural Inquiry, University College London, London, United Kingdom

**Keywords:** postfeminism, aesthetic labor, South Asia, Brazilian waxing, intimacy, beauty salon

## Abstract

Postfeminism is a neoliberal sensibility that locates femininity in the body, thereby imploring women to constantly labor on, monitor and discipline their bodies. This aesthetic labor is presented to women as freely chosen and empowering. Brazilian waxing is exemplary aesthetic labor directed at the self. Academic literature on aesthetic labor in general, and Brazilian waxing in particular, looks at white and middle-class women, as this category of women is considered the putative subject of postfeminism. Little attention is paid to racialized women from the global south who perform aesthetic labor on other women’s bodies in the global north. In this paper, I draw on my ethnographic study of two beauty salons in London run by South Asian women to argue that these South Asian beauticians are postfeminist subjects as well. The aim of challenging the putative subject of postfeminism, using the example of Brazilian waxing, is not merely to include South Asian women in the discourse, but to advance a transnational theorization of postfeminism. Such theorization, I demonstrate, leads to a better understanding of how postfeminism is implicated in global structures of power as well as the affective qualities of postfeminism including intimacy and disgust.

## Introduction

Body hair removal is considered an essential trait of femininity in many societies around the world. In countries such as the United States, United Kingdom, Canada and Australia, the obsession with removal of body hair also extends to the female pubic area, with mass media presenting it as desirable and sexy, and beauty salons offering different styles of fashioning pubic hair. Most of the studies conducted with women on pubic hair removal have been quantitative in nature, providing empirical data which sheds light on the prevalence of the phenomenon. In a study conducted with 235 white undergraduate students in Australia, it was found that at least 60% of them removed some of their pubic hair with 48% removing most or all of it, waxing being the most popular method of hair removal (Tiggemann and Hodgson, 2008). A survey of 678 majority-white British women concluded that over 80% removed hair from their pubic area, with shaving being the most used method (Toerien et al., 2005). Similarly, in a survey of 660 predominantly white women in British Columbia, it was found that 50% of the participants removed hair from the bikini line and 30% from the whole pubic area, with shaving, salon waxing and trimming emerging as popular methods (Riddell et al., 2010). While most studies have been conducted with convenient samples of white women in the global north, in a survey of 400 women in Saudi Arabia, all the women reported removing pubic hair as well, shaving being the most commonly used method (Rouzi et al., 2017).

In all these studies, women identified femininity, attractiveness and personal hygiene as the main reasons for pubic hair removal. What makes pubic hair removal different from hair removal from other parts of the body is that the pubic area, unlike arms and legs, is not normally visible to others. Therefore, the decision to remove pubic hair is orientated more toward the self and/or a sexual partner. In the survey with Australian undergraduates, “it makes me feel cleaner” was the most-cited reason for the removal of pubic hair (Tiggemann and Hodgson, 2008, 891). Cleanliness also emerged as an important theme in two qualitative studies conducted with ethnically diverse women in New Zealand and the United States, locating pubic hair as a potential site for sweat, dirt and odor (see Braun et al., 2013; Fahs 2014). Similarly, in a study conducted with Turkish-Cypriot women, the main reason for pubic hair removal was cited as comfort and prevention of odor (Muallaaziz et al., 2014). Although women acknowledge the role of societal norms, they are unwilling to attribute their behavior to beauty standards alone, framing body hair removal as a personal choice (Tiggemann and Kenyon, 1998; Li and Braun, 2016). Tiggemann and Hodgson (2008) argue that associating lack of pubic hair with cleanliness and better personal hygiene is precisely the kind of rationale that keeps women chained to a neoliberal logic of periodic consumption of products and services that stave off body hair.

The literature on pubic hair removal maps neatly on to recent literature on postfeminism and its offshoot, aesthetic labor. Although the term originated as a critique of media culture, postfeminism was soon consolidated as a cultural “sensibility” defined by certain characteristics (Gill 2007, 148). Broadly, some of the characteristics that comprise a postfeminist sensibility are the notion that femininity is located in the body and requires constant self-surveillance, monitoring and discipline; a shift from objectification to subjectification; a renewal of the idea of natural sexual difference; and an emphasis on consumerism and the commodification of difference (Gill, 2007). Thus, a postfeminist sensibility is constituted through neoliberal ideas of choice, individualism and empowerment in the service of consumerism. Both postfeminism and neoliberalism are predicated on the mobilization of an autonomous, freely choosing and agentic female subject who is required to constantly labor on and transform herself through the consumption of products and services (Gill and Scharff, 2011). Aesthetic labor or aesthetic entrepreneurship requires us all to be entrepreneurs, constantly working on our appearance—with agency and creativity—so that the project of maintaining and beautifying our bodies appears self-directed and self-managed, seemingly free of societal constraints and pressures (Gill, 2007; Winch, 2013; Elias et al., 2018).

Thus, postfeminism is a set of ideas around femininity that can be studied (Riley et al., 2017). Women strive to be rendered intelligible through these ideas, therefore aspiring to postfeminist subjecthood. The normative subject of postfeminist discourse has been posited as young, white, heterosexual, middle-class, able-bodied and conventionally attractive (Butler, 2013). Postfeminism centers an “affluent elite,” one who can access the pleasures and lifestyles associated with it, thereby occupying a white and middle-class subject position by default (Tasker and Negra, 2007, 2). Therefore, most scholars working on postfeminism have assumed white, middle class, heterosexual women as the sole subjects of postfeminist discourse.

In this paper, using the example of Brazilian waxing, I aim to complicate the understanding of the putative subject of postfeminism. More specifically, drawing on my ethnography of two beauty salons run by South Asian migrant women in London, I illustrate that the racialized women performing aesthetic labor on other women’s bodies are postfeminist subjects as well. In doing so, I demonstrate what theorizing postfeminism as transnational might look like. I also argue that focusing on the experiences of those who perform aesthetic labor on other women for a living leads to a better understanding of the affective qualities of postfeminism. I use postfeminism as a sensibility or a discourse throughout rather than an analytic perspective.

I use the example of Brazilian waxing as it is exemplary postfeminist behavior or aesthetic labor directed at the self. In discussing postfeminism, Rosalind Gill (2007, 153) writes about “phenomena such as the dramatic increase in the number of women having Brazilian waxes” and Simidele Dosekun (2015, 966) mentions “Brazilian waxes” as a kind of postfeminism that sells internationally alongside “Beyonce” and “boob jobs”. It is one of the many methods available to women for pubic hair removal. Being hairless in the pubic area as an adult woman requires work, time and money; and waxing as a method of hair removal is especially time-consuming, painful and expensive as compared to do-it-yourself methods such as shaving and trimming. What makes waxing a popular method of hair removal is that it can be performed by a professional in a beauty salon and ensures hair-free skin for a longer period, reducing or thinning hair growth with continued usage. There is, however, a mind-boggling array of styles when it comes to pubic hair waxing. The nomenclature is often confusing and used interchangeably depending on the local context. Three well-known styles of pubic hair waxing are Bikini wax, Brazilian wax and Hollywood wax. Bikini wax, as the name suggests, removes any hair that can be seen while wearing a bikini or a swimsuit. Brazilian wax takes off all the pubic hair from the front and back, leaving a small strip on the mons pubis in a shape of one’s choice. Hollywood wax, on the other hand, removes all hair completely from the pubic area. For the purpose of this paper, I will use the generic term Brazilian waxing to denote full pubic hair waxing, although “full bikini waxing” is commonly used by the South Asian participants in my research in London.

## The Transnationality of Aesthetic Labor

Writing ten years since she first conceptualized postfeminism as a sensibility, Gill (2017, 609) claims that postfeminism is now so “hegemonic” that it operates “as a kind of gendered neoliberalism” that is “difficult to recognize as a novel and distinctive sensibility.” This hegemonic form of gendered neoliberalism, however, is not a culturally or geographically specific phenomenon but is simultaneously “discursive, ideological, affective and psychosocial” (Elias et al., 2018, 25). As a cultural sensibility or discourse, “postfeminism can travel through complex social terrains, deftly adapting to cultural, economic, and political shifts while maintaining its core characteristics” (Butler, 2013, 45).

In that sense, it would be a mistake to treat postfeminism as an exclusively “Western” phenomenon (Gwynne, 2013). Although the central figure imbricated in a postfeminist discourse is white and middle-class, this does not mean that non-white and non-middle-class women are unaffected by postfeminism, but that their relationship to the pervasive cultural phenomenon remains undertheorized (Butler, 2013). One of the most important developments in literature on postfeminism in the last ten years has been an attempt to advance an intersectional analysis of the concept by incorporating race, class, sexuality and transnationality (Gill, 2017). Dosekun (2015, 966) argues for taking a transnational approach to postfeminism by acknowledging that the culture affects not only women in the West but also elsewhere. She posits that irrespective of their geographical location, a postfeminist subjectivity is available to feminine subjects with the “material, discursive and imaginative capital to access and to buy into it.” In other words, it is class and not race or geographical location that determines who gets interpellated as a suitable subject of postfeminism. Dosekun bases her argument on her research with well-educated and upper-class women in Lagos, Nigeria who engage in highly laborious and sometimes painful aesthetic labor, justifying it as self-pleasing, autonomous and empowering, thereby fashioning themselves as postfeminist subjects.

Even when class is taken into account, what gets elided in the discourse on aesthetic labor is that most women who are considered to be performing aesthetic labor are actually aesthetic consumers. These aesthetic consumers normally pay other women to perform aesthetic labor on their bodies. The beautician or therapist who performs this labor often comes from a working class, migrant and ethnic minority background. In academic literature, she is often constructed in opposition to the white, middle-class postfeminist subject. While consumers seek personal and professional advancement through the performance of aesthetic labor on their bodies, the beauticians doing this labor “work in abject conditions themselves” (Orbach, 2017, viii). As a result, although we know that beauticians work hard in low-paid jobs, sometimes under exploitative conditions, we do not know if they too reap the purported benefits of postfeminism. Aesthetic laborers are conspicuously absent from the literature on postfeminism.

What does it mean, then, to take an intersectional and transnational approach to postfeminism? A transnational approach to postfeminism must involve decentering the discourse from the global north by not thinking of it as uniquely or authentically Western culture, such that we already see it as impossible or imitative elsewhere (Dosekun, 2015). To me, however, the bigger question is that to what end do we seek to “include” women from the global south in a discourse that seemingly excludes them when the discourse itself is an object of feminist critique? The end cannot be inclusion in and of itself. The aim of an intersectional and transnational approach should not be to provide an evidence of postfeminist sensibility in non-Western cultures and countries, thereby “reinstating their otherness” but to produce an analysis of complex interconnections and power asymmetries that affect the global flow of postfeminism (Riley et al., 2017, 8).

Many women working as aesthetic laborers in the global north are racialized migrants from poorer countries in the global south (Wolkowitz, 2002). Thus, aesthetic labor is always already situated in the processes of globalization, or “the global circulation of goods, ideas and peoples” (Boris and Parreñas, 2010, 9). Although women performing labor on other women’s bodies are transnationally mobile subjects, due to lack of research in this area we have little idea if they have access to the same postfeminist discourse as their customers. What has also been overlooked by researchers in this context is that as workers trained in beauty practices and treatments, aesthetic laborers are very likely to perform aesthetic labor on their own bodies. That only middle and upper-class women buy into a discourse of wanting to be beautiful and hairless is a rather simplistic view to take. However, researchers have hitherto not focused on whether a non-middle-class, non-white beautician can be interpellated as a postfeminist subject, through her performance of aesthetic labor (on herself and others) as well as through the intimacy she shares with her customers who are putatively postfeminist subjects.

By focusing on aesthetic laborers, we can also enhance our understanding of the affective qualities of postfeminism. So far, the affective qualities of postfeminism have been understood as the affects that accompany the ideas endorsed by postfeminist culture—the disavowal of unpleasant emotions such as insecurity, anger and complaint in favor of feelings such as positivity and confidence (Gill, 2017). There is a whole gamut of emotions, however, that can only be understood as belonging to the postfeminist repertoire by extending its subjecthood to women who perform aesthetic labor on other women’s bodies. This kind of labor involves embodied and affective interactions (Boris and Parreñas, 2010). Scholars researching the sociology of the body and work have variously theorized this kind of labor as affective labor (Hardt, 1999), bodywork (Wolkowitz, 2002) and intimate labor (Boris and Parreñas, 2010). To use Carol Wolkowitz’s (2002, 497) definition of bodywork, this kind of labor “takes the body as its immediate site of labor involving intimate, messy contact with the (frequently supine or naked body), its orifices or products through touch or close proximity.” It involves touch, bodily and emotional closeness, and knowledge of personal information. It is stigmatized by the presence of bodies and dirt, and often entails the management of emotions. It involves pleasure and emotional intimacy, but it also evokes disgust.

Thus, by bringing racialized women from the global south into the fold of postfeminism, this article seeks to underline the global structures of power that aesthetic labor is implicated in as well as the affective qualities that enable this labor.

## Methods

As part of my doctoral study, I am conducting ethnographic research in two salons between 2020 and 2022: Noor’s Hair & Beauty and Maya Hair & Beauty. All the names used in this article including the names of the salons are pseudonyms. The overall aims of the study are to investigate what kinds of intimacies are formed in a beauty salon, and how does intimacy deal with differences such as ethnic, religious, class-based and sexual?

The two salons in my study are located in neighbourhoods populated by British-South Asian communities. Using business review aggregators such as Google and Treatwell, I compiled a list of salons that cater to South Asian women in different neighbourhoods in London and approached them. Noor was the first to grant me access. Since Noor’s Hair and Beauty was owned, staffed and frequented by Muslim women of Pakistani origin, I made a conscious attempt to look for a second salon that would differ in attributes so as to provide a useful point of comparison. Noor’s Hair & Beauty is a mid-range salon, owned and run by Noor (42), who came to the United Kingdom from Pakistan twenty years ago. At the time of writing this paper, she was assisted in the salon by Sara (40) who shared her migration trajectory. Noor’s customers are Muslim women of Pakistani origin, white women and a small number of black women, from both middle-class and working-class backgrounds. Maya Hair & Beauty, on the other hand, offers more affordable rates. It is owned by Anita (38), who came to the United Kingdom from Nepal twelve years ago. At the time of writing this paper, she was assisted by Rekha (40), Bharti (40) and Jaya (45) from India who had all been in the United Kingdom for less than five years. Their customers are recent migrants from India, Nepal and Sri Lanka, many of them working-class.

The methods used in my research are participant-observation and longitudinal interviews. I conduct participant-observation at the beauty salons by working as an assistant. My job largely entails sweeping the floor, making tea and apprising customers of Covid regulations. While I made sure at the outset that the salon owners and assistant beauticians understood what my research was about, I also continually remind them of my researcher role. In terms of my positionality, I believe that insider or outsider are not fixed but unstable and ever-shifting positions, differentially experienced and expressed in the field (Naples, 1996). The participants in my study and I share the same gender and race. Like them, I am also a first-generation migrant in the United Kingdom from a South Asian country and therefore share their unique vantage point of being “in-between” two cultures and societies. I can speak or follow the languages that they speak in (Hindi, Urdu, Gujarati and Punjabi). Therefore, while I have developed friendly relations with most of the participants, I am also aware that the social and cultural capital I have accrued through my education and work over the years is visible in my comportment, accent and clothes, marking me as an outsider, in addition to my marital status (or lack thereof) which is unusual for an Indian woman of my age.

My research follows an “inductive-iterative” approach wherein ethnography moves back and forth between theory and analysis, data and interpretation (O’Reilly, 2009, 104). I went to the field with an open mind and relied on my field notes for themes to emerge. I was compelled to write this paper after recording the affective reactions of both the beauticians and the customers to Brazilian waxing and feeling highly inquisitive about it as I have never done it myself and am held back by a feeling of shame. In the first part of the next section, I present two vignettes from participant observation at Maya Hair & Beauty. The two vignettes have been chosen to demonstrate the range of feelings that beauticians might experience while providing a Brazilian wax to their customers and the kinds of relations those feelings can lead to. In the second part, I draw on semi-structured interviews with beauticians at both the salons where I asked them questions about Brazilian waxing such as when did they first learn about the service, their experience with customers and their own hair removal habits.

## Observations From the Field

### Vignette 1

Maya Hair & Beauty is located on a busy high street in London dotted with a temple, Indian grocery shops and eateries. The salon itself is quite small and cramped. It consists of a low-ceilinged room with mirrors, three salon chairs and two benches for customers. The walls are covered in cheap blue wallpaper and the room does not have a separate reception area. There is a massage bed in one corner of the room, partitioned off with a curtain, where a customer can lie down for a private treatment such as massage, facial or body hair waxing. Despite the lack of space, the salon is always clean and organized. There is a small temple with Hindu deities affixed to the wall close to the entrance. On one of the shelves in the wall stands a small transistor radio that is always set to Sunrise, a South Asian FM channel. The latest Bollywood hits can be heard in the background while getting a beauty treatment at Maya Hair & Beauty.

Bharti, who works at the salon two days a week, has just finished giving a full-body massage to a customer. Late afternoon, Joanna, a young Public Relations professional from India walks in. It is her first time at Maya Hair & Beauty. She asks for a hair spa and a Hollywood wax and is assigned to Bharti. Joanna begins conversing with the staff in English. “You don’t like Hindi?” Bharti, a native Gujarati speaker asks her while massaging her head, encouraging her to speak in Hindi. Once Joanna starts speaking in Hindi, Bharti and she do not stop chatting for the next 3 h. Bharti massages Joanna’s hair with oil, applies a protein mask, steams it and then washes it. In the time this takes, they discover that they had both arrived in the United Kingdom from India two years ago following their husbands, had a rough first year when they both wanted to leave, and finally got acclimatized to London. Bharti asks Joanna about her job. When she answers with “Public Relations,” Bharti does not understand. Joanna simplifies it for her by saying that she writes for corporate clients. Bharti tells her that she left behind her two children (who are being looked after by her mother and mother-in-law) and her beauty salon in India because her husband believed that the United Kingdom had better job opportunities.

After all this is done, Bharti asks Joanna to lie down on the massage table in the curtained enclosure. The sound of their chatter and giggles travels out of the curtain to the main area of the salon where I am seated. Three hours go by. Finally, when they come out into the main area of the salon, Bharti offers Joanna some fresh orange juice she had got from home that morning. Joanna advises her to “train” her husband to cook and do household chores the way she has trained hers, and Bharti asks her if she can have her phone number for more advice.

I ask Joanna if I can interview her. The following day, she and I meet over a video call. I ask her why she got a Hollywood wax. Although she insists that it was on account of personal hygiene, she also mentions “feeling sexy” after hair removal. When I ask her to describe her experience with Bharti, she says: She didn’t judge me for anything. For anything. In fact, whatever I shared with her, she seemed to reciprocate the same. Like she would tell me an experience of hers… something that matched that part […] you know after the waxing she was like, “Come on then, off you go.” And I was like, “So are you going to give me a tissue or anything” because they generally give you a tissue. She was like, “No, you are clean, that's fine.” She generally mentioned that a lot of people who come here, their hygiene is quite poor. And then I asked her, you know, “Do you feel a bit grossed out when you do things like these” and she said, “Do you think doctors feel grossed out when they help a woman give birth to a baby?” I said, “Not really but they are doctors.” And she said, “We do the same thing. We don’t help you have a baby but we make you pretty down there.”

When I mention to Bharti later that I noticed that she and her customer were having a full-fledged conversation and that she was letting her in on her personal life, she says: “…the customer should feel that she is sharing her story with me as well as listening to mine. When it’s a friendship, there is no such thing as “professional”! She is talking to me because she considers me a friend. If she thought of me as a professional, why would she talk to me like that?”

### Vignette 2

A middle-aged Indian woman comes in for her scheduled appointment at Maya Hair & Beauty. Rekha and Jaya take turns to service her as she has booked a long list of treatments. She is taken to the tiny enclosure for a Brazilian wax first. Rekha volunteers to do it as Jaya joined the salon only a week ago. About half an hour later, she comes out retching. She gestures with her fingers at the other beauticians and I to show how long the customer’s pubic hair were—about two inches long is what I comprehend from the distance between her thumb and her index finger. She complains about the customer’s poor hygiene and vaginal odor. What seems to have disgusted her even more is that the woman asked her to take a picture of her shaved pubic area on her phone to send her boyfriend. Rekha does not want to service the customer any longer, so Jaya goes in. She is much more sympathetic to the customer and pampers her including taking videos of her for her boyfriend. On leaving the curtained enclosure, the customer announces to everyone present in the salon that she got her pubic area waxed in preparation for a rendezvous with her boyfriend. The announcement is met with awkward silence and purposeful glances.

Jaya proceeds with the customer with extraordinary patience, alternating between humouring her and scolding her like a child to sit still and keep her phone aside. She goes on to thread her face and then gives her a pedicure. Toward the end of their session, the customer opens a packet of crisps and feeds Jaya with her own hands while Jaya is working on her toenails. They even give each other nicknames. Before leaving, the customer tips both Rekha and Jaya generously. Apparently, she did not catch wind of Rekha’s aversion and disgust toward her. She also takes Jaya’s number after asking if she would be open to giving her a body massage at home.

## Interviews

When talking about Brazilian waxing, all the participants in my study used the cut-and-dried language of professionalism. They resorted to clinical language about Brazilian waxing, some comparing themselves to doctors, as seen above, displaying a matter-of-fact attitude about work that must be done without letting feelings of discomfort or embarrassment get in the way. Moreover, they emphasized the importance of wearing gloves while doing it. As Anita said: “We have to protect ourselves first.” Here, the “protection” is against customers with poor levels of hygiene and vaginal odor, demonstrating that despite the employment of a professional attitude and clinical language, disgust is a major factor in Brazilian waxing. As Rekha said: “[I wear] gloves and mask. This mask thing has started now but we used to wear it even before. Some people (lowers voice) it smells bad…”

For Noor, gloves are non-negotiable. She said: “It’s compulsory for me to wear gloves. I cannot do it with bare hands […] it is someone’s private part. I do not know how it is, whether it is clean or not, why should I touch it? It’s a matter of hygiene.”

Bharti said that she did not wear gloves while doing bikini wax in India, but she learnt about it in the United Kingdom. She said: “It’s better for the customer and for me. But in India they do not teach this. I am telling you the truth. When I go back, I will tell everyone that you must wear gloves.”

With respect to themselves, all but one of the beauticians said that they regularly removed pubic hair with “bikini wax” being their preferred method of hair removal. The one beautician that did not use this hair-removal method claimed that she naturally did not have any pubic hair but certainly would have waxed if she did. Nearly all of them made a reference to cleanliness and hygiene while describing why they choose to get a Brazilian wax. Although all of them are in heterosexual marriages, none mentioned their partner playing a role in their decision. This contrasted with what they believed to be the reason why their customers got a Brazilian wax.

Bharti was clear in making this distinction when she said: “The girls here get it done for husbands or boyfriends. In India also, women get it done on their wedding. They want that area to be clean… you know? (giggling) And if I speak about myself, I get it done because I like it clean.”

Noor mentioned religion as a factor, in addition to cleanliness. She said: “We Muslims have to do a clean-up every month… after period, before prayers… we have to. We have to take off hair from arm pits and the pubic area.”

Bharti, who ran her own beauty salon in India for five years before coming to London in 2018, also described how she was reluctant to do Brazilian waxing at the start of her career. What marked the transition for her is when she tried it for herself and was amazed at the results. “When I did it for myself, then I realized that it feels clean, so I must give this experience to others. Initially I did not do it for one year. I did not do it myself and I did not do it for customers […] Then the woman I learnt from, my aunty, she asked me to try it. She said it is nice and feels very clean. There will not be any growth for two months. Then I tried it and I liked it. So, I felt I should tell the customers also that this is nice. Now I tell everyone how nice it is,” she said.

Rekha, who worked in a beauty salon in a small town in India before moving to London in 2016, started doing Brazilian wax for customers in the United Kingdom. It was not common in the Indian region she lived and worked in, except for brides. When she started going to a beauty school in London, she learnt about the benefits of Brazilian waxing. “One must keep the bikini area clean. Now I am studying […] and it figures in that as well… the cleaner you keep that part, the more hygienic it is for you,” she said.

She, too, tried it on herself before she started doing it for her customers: “I did not use to do it for myself. I used to shave. Then one time I tried it and oh my god, the pain..! I was dancing with pain. But that was because it was my first time. Now I do it on myself […] I thought all these women who get it waxed, it must hurt them? Let me see for myself how much it hurts […] Whatever you are doing as a beautician, you must have experienced it on yourself, especially when it is painful.”

All the participants dismissed my suggestion that Hollywood waxing (complete hair removal from the pubic area), for which they use the term “full bikini waxing,” was a relatively new trend in beauty. They were particularly keen to emphasize how it was not something that was only sought after in London but was a common “treatment” in parts of South Asia they came from. As Bharti said: “Even people from the villages [get it done]. The difference is that people know the language here. They call it Hollywood here and bikini waxing there. But everybody knows what it means.”

Anita, who started as a beautician in her sister-in-law’s salon in Nepal about twenty-one years ago, said that she knew about bikini waxing since the beginning of her career. Comparing the trend in Nepal to India, she said: “[Girls] are more advanced there. Even young ones come for bikini waxing. Saying they have boyfriends.”

Noor, who forayed into beauty about twenty-five years ago in her aunt’s salon in rural Pakistan, said: “[…] even in Pakistan, in the posh areas, this was done since a long time. But people like us… we are from the middle class...we did not know about it. But my aunty had a salon, so I knew. I had seen customers who came for this. Brides came in and we did bikini waxing for them. But the trend is not new, I think it is old […] at least since I have been in business […] twenty-five years.”

## Discussion

### Postfeminism as Transnational

#### The Postfeminist Narrative

From the vignettes and the beauticians’ narratives above, two reasons emerge for why women get a Brazilian wax: personal hygiene/cleanliness and sexual attractiveness. The beauticians often lay claim to the former while attributing the latter to their customers, using it as a device to articulate themselves as more independent and autonomous subjects who wax their pubic hair to please themselves rather than their sexual partners. This sentiment is best captured when Bharti said: […] “And if I speak about myself, I get it done because I like it clean.” As I mentioned before, although all the beauticians are married, none of them mention their partners and their preferences while discussing why they opt for a Brazilian wax. Instead, they accept and perpetuate the idea of a hairless vagina as hygienic. As Rekha, who learnt about the benefits of Brazilian waxing in a beauty school in London said: “With the bikini area, the cleaner you keep it the better. Then there is less risk of infection.”

The rationale of hygiene, however, is as much steeped in a neoliberal and postfeminist sensibility as sexual attractiveness. It is also a rationale that helps women uphold Brazilian waxing as an autonomous “choice.” Pubic hair removal as a form of bodily modification originated in a specific cultural moment in history but has now become so commonplace that its relation to hygiene, even though not supported by any medical evidence, seems to have common sense appeal. In fact, medical evidence suggests the contrary: pubic hair serves its own hygienic purpose by trapping bacteria and other pathogens and preventing them from entering the vaginal opening (Hoffman, 2016; Craig and Gray, 2019). Hygiene, when pertaining to the vagina, often refers to subjective feelings of cleanliness rather than objective indicators of women’s health. Hygiene as socially constructed is also evidenced by the growing market of vaginal hygiene products for women vis-à-vis the lack of hygiene products geared toward men (Jenkins et al., 2018).

The second reason, i.e., sexual attractiveness, is much easier to deconstruct. Postfeminism dictates that women take responsibility for producing themselves as desirable heterosexual subjects as well as pleasing men sexually (Gill, 2007). In this psycho-sexual regime, getting a Brazilian wax can be framed in terms of taking ownership of one’s sexuality, a project that will also lead to a better sexual experience for the male partner. In tracing the psychic life of postfeminism, Gill illustrates the “psychological complexity” of how power works “by structuring our sense of self, by constructing particular kinds of subjectivity so that socially constructed ideals of beauty or sexiness are internalized and made our own” (Gill, 2007, 76).

Now this is not to dismiss that women may actually feel cleaner and/or sexually attractive after getting a Brazilian wax. This is also not to imply that women are subservient to cultural norms and media-messaging to such an extent that they blindly internalize the messages that are passed on to them. But since the agency vs. structure debate falls outside the remit of this paper and has been discussed in academic literature widely anyway, what I am interested in and fascinated by is women’s adoption of a highly painful and expensive method of pubic hair removal and the homogeneity of the narrative supplied in its favor across cultures. In different societies where women have been asked about why they get a Brazilian wax, they have mentioned personal hygiene and sexual attractiveness, presenting it as a free, autonomous and informed choice. It is imperative to a postfeminist sensibility that one’s practices, no matter how painful or harmful, be presented as freely chosen, even as indulgence (Gill, 2008). This elucidates how postfeminist narratives travel in a globalized world, especially as it relates to the participants in my study who are migrant women from South Asia working as aesthetic laborers in the United Kingdom. However, I expound in the next section why some amount of caution must be exercised in taking personal hygiene and sexual attractiveness as indicators of a postfeminist sensibility across cultures.

#### The Pitfalls of Assuming Postfeminist Homogeneity

As most of the studies on pubic hair removal (quantitative or otherwise) have been conducted with women from the global north, it creates a false impression of Brazilian waxing being an exclusively Western phenomenon. A transnational theorization of postfeminism, however, must also caution us to the dangers of homogenization of women in the non-Western world. For example, pubic hair removal has been widely practised in Muslim cultures around the world, in a context that cannot be easily attributed to postfeminism. The rules of ritual purity in Islam specify removal of pubic hair at least once every forty days since the onset of menarche (Rouzi et al., 2017). Women in non-Western cultures draw upon discourses other than postfeminism with similar modalities and results. In a systematic, cross-cultural analysis of academic literature on pubic hair removal practices in non-Western cultures, it was found that women cited hygiene as the primary motivator, corresponding with findings in Western cultures (Craig and Gray, 2019). In an ethnographic study of Muslim women in lower-income neighborhoods of Cairo, the researchers found that women remove pubic hair using a homemade paste of lime, sugar and water and consider it essential to the production of married femininity and cleanliness (Malmström, 2015). They consider body hair, especially pubic hair to be dirty and its depilation necessary in order to pray.

It is also noteworthy that among the two studies previously mentioned in this article that were conducted with Muslim women, only 5.5% of the women surveyed in Saudi Arabia mentioned Islam as a reason for pubic hair removal (Rouzi et al., 2017). Similarly, only 8% of the Turkish-Cypriot women attributed public hair removal to Islam (Muallaaziz et al., 2014). The majority of women in both these studies cited reasons such as hygiene, appearance and prevention of odor. Thus, it is clear that Muslim women living in the non-Western world put in considerable aesthetic labor into maintaining a hairless pubic area in the service of hygiene and femininity. However, to ascertain whether these practices can be theorized as postfeminist or not needs a careful analysis that is attuned to cultural and contextual specificities such as class, age, generation and the method of hair removal. For example, the hair removal practice of working-class middle-aged women from lower-income neighborhoods of Cairo using homemade wax might not be amenable to the same analytic framework as the hair-removal practice of middle-class young women in Jeddah who opt for a Brazilian wax in a beauty salon. Although the discourse of postfeminism suffers from a homogenizing imperative that erases the differential experiences of women, scholars researching postfeminism must steer clear of it in the interest of a nuanced and context-specific analysis.

As for the two Muslim participants in my study, while pubic hair removal in and of itself was constructed as a religious mandate, Brazilian waxing was articulated as a postfeminist choice. The participants seamlessly combined religious obligations with their knowledge of beauty work illustrating how postfeminism might build upon other discourses without conflict. Noor emphasized that girls must start getting a Brazilian wax at the onset of puberty. When I asked her why one must choose Brazilian waxing over other hair removal methods, she said: “The benefit it is that it stops your hair growth. You skin is clean. It does not get dark. The hair becomes very light gradually. Even my clients, ones who started Brazilian waxing with me, ten years ago, their hair is almost non-existent now. It also comes off easily … And it is good, no? You feel nice and clean.”

### Affective Qualities of Postfeminism

#### Unpleasant Feelings: Disgust

A distinct affective register forms an integral part of postfeminist sensibility. Postfeminism offers individual solutions to structural problems couched in the language of empowerment, choice and self-responsibility. Rosalind Gill and Shani Orgad (2017) have written about the pervasive “confidence culture” wherein the source of women’s problems is located in lack of confidence and self-assuredness. Thus, women are told that by changing their behavior, they can overcome their problems and transform their lives. As part of this culture, confidence is depicted as the “new sexy” while insecurity or lack of confidence as “undoubtedly the new ugly” (Gill and Orgad, 2017: 27).

As a corollary to “confidence culture,” studies have shown how women attach the feelings of self-consciousness and shame to the presence of pubic hair (Jenkins et al., 2018). Thus, getting a Brazilian wax helps them to overcome bodily shame and insecurity and feel confident and attractive. Postfeminism plots a rather simple trajectory of feelings for its subjects with the capacity to buy “confidence” through the labor of other women. But what about the feelings of aesthetic laborers? What unpleasant feelings do they experience and more importantly, manage in order to enable their customers to overcome those unpleasant feelings repudiated by postfeminism?

This quote by Noor sums up the emotions experienced by women performing the aesthetic labor of Brazilian waxing on other women succinctly: “When they not clean, Oh my god, I feel angry—why did I agree to it? What should I do? Should I just leave it halfway? You know… They will come in all decked up … hair straightened, makeup on, perfume on… and then they are dirty down there.”

The feeling of disgust is related to the degree of intimacy between people; the less intimate people are, the more intense the level of disgust elicited by certain factors such as body products that are seen as possible sources of contamination (Buzekova and Isova, 2010). In the case of intimate contact necessitated by Brazilian waxing, disgust is evoked by vaginal discharge and fecal residues, as told to me by the participants in my research. In addition to what is deemed as poor hygiene, disgust can also be evoked by “inappropriate” sexuality (Buzekova and Isova, 2010). In the second vignette, Rekha is disgusted not just by the customer’s lack of hygiene but also her unflinching admission of non-marital sexuality and playfulness. This feeling of disgust disrupts both physical and emotional intimacy, as Rekha finds herself unable to service the customer and leaves the enclosure. Jaya, on the other hand, succeeds in overcoming disgust and establishes both physical and emotional intimacy with the same customer.

Disgust is a common emotion experienced by beauticians toward customers who are deemed unclean and unhygienic. A common thread that runs through the narratives of the participants in my study is how they did not want to perform Brazilian wax as soon as they entered the profession but took some time to prepare themselves for it. Even in the second vignette, Rekha volunteers to do Brazilian waxing in order to spare Jaya the trouble because she is new to the salon. Beauticians rely on a medical discourse and distancing techniques such as gloves to overcome the negative feelings associated with intimate waxing. This is also evident in the first vignette where Bharti switches between contradictory frameworks to make meaning of her interaction with Joanna: while she views their overall interaction as a “friendship” marked by reciprocity, she resorts to medical language when referring to Brazilian waxing where her job is analogous to that of a doctor. Besides acting as a barrier against disgust, distancing techniques have a symbolic meaning that goes beyond the rationale of hygiene. They signify a shift in the way pubic hair removal is perceived: from low status, gendered and sexualized labor to a professional service provided by a trained expert. The few customers I interviewed emphasized how the beautician wearing gloves puts them at ease as well. Distancing, then, is necessary in order to get close. Disgust and intimacy are not dichotomous affective qualities; overcoming disgust is a prerequisite for building intimacy.

Therefore, the affective register of postfeminism must include not just the feelings that a proper postfeminist subject ought to have, but also the ones that she must manage in order to get there. By emphasizing the experiences of aesthetic laborers, we become attuned to the visceral feelings around privacy, touch, odor and disgust which do not make it to the glossy magazine pages announcing the latest trend in pubic hair waxing. It also alerts us to the presence of unpleasant feelings of aesthetic labourers such as disgust and anger, in addition to the insecurity of aesthetic consumers, that must be managed in the process of making “confident” postfeminist subjects.

#### Pleasant Feelings: Postfeminist Sisterhood or Intimacy

In what might seem paradoxical to the individualism peddled by postfeminism, Alison Winch and Akane Kanai trace the affective modes of belonging to a postfeminist sisterhood in popular culture and blogs (Winch, 2013; Kanai, 2017a; Kanai, 2017b). It is, however, not contradictory because this affective relatability does not displace the focus on individualism but fosters a sense of belonging based on postfeminist sameness (Kanai, 2017b). Postfeminist sisterhood draws on assumptions of normative feminine homogeneity. Here, feminine sociality becomes a way of reinforcing gender normativity in terms of beauty standards and consumption patterns. Girlfriends share similar feminine consumption patterns and beauty standards whereby the role of girlfriends is also to discipline each other in terms of bodily normativity (Winch, 2013). This sisterhood, however, is not without its affective pleasures or a “collective fantasy of togetherness” that sidelines differences of class and race (Kanai, 2017a). Postfeminism dictates that one can gain membership to a sisterhood based on postfeminist practices irrespective of the race and class they belong to.

A beauty salon is an intimate space based on a similar kind of postfeminist sisterhood predicated on normative feminine bodily and aesthetic practices. Since the customers at Maya Hair & Beauty are mostly the same race as the beauticians, I will restrict my comments here to class and affect. Existing literature reproduces a linear narrative about class with respect to postfeminist subjectivity. Because the putative subject of postfeminism is middle-class, the working-class woman is presumed to be an aspirational subject who wants to transform herself with the help of class-privileged experts in order to acquire social and cultural capital (McRobbie, 2008). The beauticians in my research, then, complicate the narrative of class and postfeminism as “working-class experts.” With regards to their class-privileged clients, the beauticians harbor a sense of identification and sameness by focusing on their common experiences. As seen in the vignette about Bharti and Joanna, the two women, despite the difference in their educational attainment and income, bonded over their shared migration trajectory to the United Kingdom and common experiences including, but not limited to, their pubic hair removal practices. Relatability with clients is also visible in both Bharti and Rekha’s framing of Brazilian waxing as something they started doing for customers once they realized its benefits for themselves.

Positioning themselves as experts in their field and as friends to their customers also helps beauticians to alleviate the difference in social status between themselves and their clients (Gimlin, 1996). Thus, in the case of working-class aesthetic laborers, class is not an impediment in claiming postfeminist subjectivity. Beauticians lay claim to postfeminist sisterhood alongside their customers by leveraging their specialized knowledge, professionalism and their understanding of the pain of procedures such as Brazilian waxing. It is worth reiterating, however, that overcoming disgust and aversion is a prerequisite for the beauticians to build intimacy and sisterhood with their customers.

## Conclusion

While I agree with Dosekun that postfeminism in the global south is available to class-privileged women, I have argued in this article that it is not exclusively available to class-privileged women only. When thinking of postfeminism together with transnationality, we tend to assume that the imagination for transnational mobility and access to it is the preserve of class-privileged people only. In reality, it is both the rich and the poor who migrate from the global south to the global north in search of better incomes and lifestyles. A transnational approach to postfeminism, then, must take into account the bigger picture: how care deficit in the global north leads to migration of women from the global south who take up low-paid jobs including aesthetic labor. Aesthetic laborers also emerge as a special category of workers who can compensate for their class position by foregrounding their professional knowledge and sameness of experiences with their class-privileged customers.

The aim of showing that the South Asian women working as beauticians in London are postfeminist subjects as well is not merely to claim inclusion for them in the postfeminist project but to situate their postfeminist narratives in a larger socio-political context. All the beauticians in my research migrated to the United Kingdom in search of better incomes for their families. All of them had worked in beauty salons in the countries they came from and started working in beauty salons run by other South Asian women upon arriving in London. This is because jobs in the beauty industry have low entry barrier as they are considered low-skilled and are low-paid. As a result, part-time beauticians such as Bharti and Jaya have to work multiple jobs to make ends meet. When her shifts at Maya Hair & Beauty were reduced to two days a week following the aftermath of the COVID-19 pandemic, Bharti took up a job that entailed caring for an old Indian couple with Parkinson’s for five days a week. Jaya, who arrived in London amid the pandemic started cooking in an Indian fast-food outlet after failing to find a full-time job in a beauty salon.

In this paper, I have shown that the South Asian women working as beauticians in London who participated in my study subscribe to postfeminist narratives of individual agency, empowerment and hygiene vis-à-vis Brazilian waxing. While foregrounding the similarity of their experiences with their customers, they also rely on distancing techniques such as use of medical discourse and gloves in order to manage the unpleasant feelings arising from intimate encounters with their bodies. A transnational approach to postfeminism, then, also contributes to our understanding of the affective qualities of postfeminism. By focusing on the experiences of beauticians, we understand disgust as an emotion that needs to be managed in the process of esthetic labor. Once disgust is effectively managed, an intimacy or sisterhood based on normative femininity can be fostered between the beautician and the customer. Therefore, looking at transnational aesthetic laborers as postfeminist subjects is crucial to expanding our understanding of postfeminism.

## Data Availability

The original contributions presented in the study are included in the article/Supplementary Material, further inquiries can be directed to the corresponding author.
